# Establishing a framework for best practices for quality assurance and quality control in untargeted metabolomics

**DOI:** 10.1007/s11306-023-02080-0

**Published:** 2024-02-12

**Authors:** Jonathan D. Mosley, Tracey B. Schock, Chris W. Beecher, Warwick B. Dunn, Julia Kuligowski, Matthew R. Lewis, Georgios Theodoridis, Candice Z. Ulmer Holland, Dajana Vuckovic, Ian D. Wilson, Krista A. Zanetti

**Affiliations:** 1 Center for Environmental Measurement and Modeling, Environmental Protection Agency, Athens, GA 30605 USA; 2 Chemical Sciences Division, National Institute of Standards and Technology (NIST), Charleston, SC 29412 USA; 3 IROA Technologies, Chapel Hill, NC USA; 4 Centre for Metabolomics Research, Department of Biochemistry, Cell and Systems Biology, Institute of Systems, Molecular and Integrative Biology, University of Liverpool, Liverpool, L69 7ZB UK; 5 Neonatal Research Group, Health Research Institute La Fe, 46026 Valencia, Spain; 6 Life Sciences Mass Spectrometry Division, Bruker UK Limited, Coventry, CV4 8HZ UK; 7 National Phenome Centre & Division of Systems Medicine, Department of Metabolism, Digestion & Reproduction, Imperial College London, London, W12 0NN UK; 8 BIOMIC_Auth, Center for Interdisciplinary Research and Innovation (CIRI-AUTH), Aristotle University Thessaloniki, 57001 Thermi, Greece; 9 Eastern Laboratory, Office of Public Health Science (OPHS), Food Safety and Inspection Service (FSIS), Department of Agriculture (USDA), Athens, GA 30605 USA; 10 Department of Chemistry and Biochemistry, Concordia University, Montreal, QC H4B 1R6 Canada; 11 Division of Systems Medicine, Department of Metabolism Department of Metabolism, Digestion and Reproduction, Imperial College, London, W12 0NN UK; 12 Office of Nutrition Research, Office of the Director, Division of Program Coordination, Planning, and Strategic Initiatives, National Institutes of Health, Bethesda, MD USA

**Keywords:** Untargeted metabolomics, Reproducibility, Guidance, Quality assurance (QA), Quality control (QC), Liquid chromatography–mass spectrometry (LC-MS)

## Abstract

**Background:**

Quality assurance (QA) and quality control (QC) practices are key tenets that facilitate study and data quality across all applications of untargeted metabolomics. These important practices will strengthen this field and accelerate its success. The Best Practices Working Group (WG) within the Metabolomics Quality Assurance and Quality Control Consortium (mQACC) focuses on community use of QA/QC practices and protocols and aims to identify, catalogue, harmonize, and disseminate current best practices in untargeted metabolomics through community-driven activities.

**Aim of review:**

A present goal of the Best Practices WG is to develop a working strategy, or roadmap, that guides the actions of practitioners and progress in the field. The framework in which mQACC operates promotes the harmonization and dissemination of current best QA/QC practice guidance and encourages widespread adoption of these essential QA/QC activities for liquid chromatography-mass spectrometry.

**Key scientific concepts of review:**

Community engagement and QA/QC information gathering activities have been occurring through conference workshops, virtual and in-person interactive forum discussions, and community surveys. Seven principal QC stages prioritized by internal discussions of the Best Practices WG have received participant input, feedback and discussion. We outline these stages, each involving a multitude of activities, as the framework for identifying QA/QC best practices. The ultimate planned product of these endeavors is a “living guidance” document of current QA/QC best practices for untargeted metabolomics that will grow and change with the evolution of the field.

**Supplementary Information:**

The online version contains supplementary material available at 10.1007/s11306-023-02080-0.

## Introduction

Metabolites and lipids play important and varied biological roles. The comprehensive measurement of these small biomolecules, known as metabolomics, underpins many modern efforts in multidisciplinary and systems-biology research. The approach employs advanced analytical instrumentation for the separation and measurement of the constituent components of complex biological samples with the intention of revealing metabolite signatures (i.e., profiles) that provide diagnostic, predictive, or otherwise characteristic information about the biofluids, tissues, organisms, foods, etc., from which those samples are derived. The approach is widely practiced across the globe using diverse technologies and methods (Alarcon-Barrera et al., 2022; Fiehn, 2016; Patel et al., 2021; Wishart et al., 2022; Zhou et al., 2012), giving rise to a host of challenges in demonstrating the quality and reproducibility of measurements and results. Thus, accepted quality assurance (QA) and quality control (QC) practices and protocols are needed across all applications of untargeted metabolomics (as is already applied for targeted analyte assays in pharma and bioanalysis) if this promising field is to realize its greatest potential.

In practice, metabolomics is an amalgamation of several different fields including analytical chemistry, statistics, biochemistry, bioinformatics, translational medicine, epidemiology, toxicology, and regulatory practices. By necessity, practitioners of untargeted metabolomics have borrowed aspects of the QA/QC best practices from these areas to introduce rigor and reproducibility in their work. However, no specific document currently meets the needs of the untargeted metabolomics community, being either too general (International Organization for Standardization [ISO], 2015) or too specific and geared towards targeted analyses of drugs and their metabolites (Food and Drug Administration [FDA], 2001; FDA, 2018; International Council for Harmonisation of Technical Requirements for Pharmaceuticals for Human Use [ICH], 2022), or clinical biomarkers (“Standards and Certification: Laboratory Requirements”, 1988; Clinical and Laboratory Standards Institute, 2021; Tsikas, 2018). A need therefore exists to better identify QA/QC best practices for untargeted metabolomics research.

Specifically, comprehensive guidance must address key phases of experimentation including (1) the planning phase (e.g., study design, sample collection, shipping and storage, instrument maintenance, and sample preparation); (2) the data collection phase (e.g., system-suitability testing, use of QC samples and data generation); and (3) the data analysis and dissemination phases (e.g., data quality review, data mining and interpretation, metabolite identification or annotation, and data sharing) (Evans et al., 2020). Here we use “guidance” according to the Oxford definition, “Advice or information aimed at resolving a problem or difficulty, especially as given by someone in authority” (Mar 2023). Such guidance could enable researchers to obtain more reliable data and provide objective evidence of data quality through reporting (Wilson et al., 2021), ultimately allowing for comparisons across workflows, studies, and laboratories. This guidance should also be applicable to all metabolomics practitioners, regardless of discipline. Thus, it should consist of both universal best practices as well as those used in special cases, such as field sampling for environmental samples, cohorts for human studies, and longitudinal studies. To accomplish this, guidance must be capable of meeting the appropriate objectives for the analytical platform (LC-MS, GC-MS, NMR spectroscopy, etc.) and the intended purpose of the study. This “fit-for-purpose” principle is imperative for guidance to be inclusive (e.g., not all labs have the same resources and infrastructure), flexible (“context of use”, i.e., different applications require differing levels of QA/QC), and above all non-prescriptive to reach the largest contingent of practitioners (Goodman et al., 2020). Because best practices must ultimately be established by the practicing community, guidance should be subject to revision on an ongoing basis. Ultimately, the development of this information should have optimal community participation, and the results should be openly available to both new and established practitioners, including those employing untargeted metabolomics within multidisciplinary teams.

Recognizing this need, the community-led Metabolomics Quality Assurance and Quality Control Consortium was formed from a 2017 Think Tank effort comprised of international representation from government, industry and academia (Dunn et al., 2017) to identify and address the current knowledge gaps in assessing study and data quality for untargeted metabolomics studies (Beger et al., 2019). It is the intention of this consortium, and specifically the Best Practices Working Group (WG) contained therein, to promote and maintain the community’s best practices as they relate to QA/QC in untargeted metabolomics, providing a framework in which mQACC can harmonize and disseminate guidance.

## Background

Objective demonstration of the quality and reliability of untargeted metabolomics data poses unique challenges in part because of the potential breadth and unknown nature of the metabolites measured, their wide linear dynamic range of concentrations, and the presence of both endogenous and exogenous metabolites. Existing guidance for targeted assays primarily focuses on answering the question, “Does the method measure the intended analyte accurately and precisely with sufficient sensitivity and selectivity (FDA, 2018)?” However, in an untargeted context and in the absence of “targeted analytes”, the answer can only be sought *after* a measurement is made, and only then within the constraints of the analytical method used. A pertinent example here would be to compare QC samples in a targeted vs. untargeted context. In a targeted assay, QC samples are made from reference standards, where concentration ranges of analytes are known beforehand, and QC samples are evaluated in-study against acceptance criteria based on pre-determined parameters (e.g., precision and accuracy) (FDA, 2018). In an untargeted assay, one widely used type of QC sample is made from pooled aliquots of study samples (hence, pooled QC sample), so concentrations and identities of all analytes are not known comprehensively at the time of measurement (Dunn et al., 2011; Gika et al., 2007; Sangster et al., 2006). Indeed, the identities of many of the measured analytes may not be known even a long time after publication, if ever, belying the surety of analyte identities in targeted assays. Similarly, in targeted assays, range and linearity are validated using calibration curves, but such an absolutely quantitative approach is not directly applicable in untargeted metabolomics. Conversely, the use of a pooled QC dilution series can allow for defining linearity and range in a relatively quantitative context (Sands et al., 2021). Indeed, reliable relative quantification is a desirable outcome for the untargeted study. Furthermore, validation parameters like sensitivity, specificity, and selectivity cannot be evaluated ahead of time for metabolites that are unknown and/or in the absence of blank matrices. The same is true for other key issues affecting reliability, including variability, range of measurements, and stability. Of course, subsequent measurements can be informed to some extent by prior studies performed under identical experimental conditions, but from a practical standpoint, the fact remains: the retrospective nature of QA/QC for untargeted assays precludes the ability to optimize and validate the method for unknown specific metabolites before the in-study measurement occurs. Thus, the existing guidelines for targeted assays cannot apply for untargeted assays. Instead, the relevant questions are, “Does the metabolomics method measure the metabolome profile reliably?” and, “For which metabolites is this statement true?”, highlighting the important goal to have reliable metabolite annotation in the untargeted assay. Hence, untargeted metabolomics is in dire need of its own accepted guidance.

Recognizing this, the metabolomics community has been seeking best QA/QC practices even while the field continues to advance. For example, when the concept of the pooled QC sample was introduced as a possible QC approach for untargeted metabolomics (Sangster et al., 2006), more than three decades’ worth of research in this arena had already been published, albeit under varied terms (Fiehn, 2002; Nicholson et al., 1999; Nicholson et al., 1984; Pauling et al., 1971). While the pooled QC approach has repeatedly shown to be essential both to assess and improve the quality of metabolomics data (e.g., through batch correction) (Broadhurst et al., 2018; Dunn et al., 2011; Kirwan et al., 2013), recent evidence from mQACC community engagement efforts suggests that pooled QCs are still underused, despite their increasing adoption by LC-MS-based untargeted metabolomics practitioners (Broeckling et al., 2023). Similarly, the use of system-suitability testing (SST) to ensure the analytical system is fit-for-purpose has gained significant traction in the field (Broadhurst et al., 2018; Evans et al., 2020; Viant et al., 2019). However, recent mQACC activities have highlighted the lack of community-wide agreement on metrics to use or acceptance criteria to apply during SST. Indeed, an mQACC workshop held at the 2022 Metabolomics Society conference in Valencia, Spain, clearly demonstrated that there is a broad coverage of areas for which there exists a high demand for best QA/QC practices (Dunn et al., 2023). Hence, mQACC has determined that now is the crucial time both to identify and to disseminate these practices in a manner conducive to widespread adoption. It is our opinion that this course of action will allow the metabolomics community to continue flourishing by protecting itself from erroneous and/or unreliable results that could potentially tarnish the field.

### Scope

**Fig. 1:**
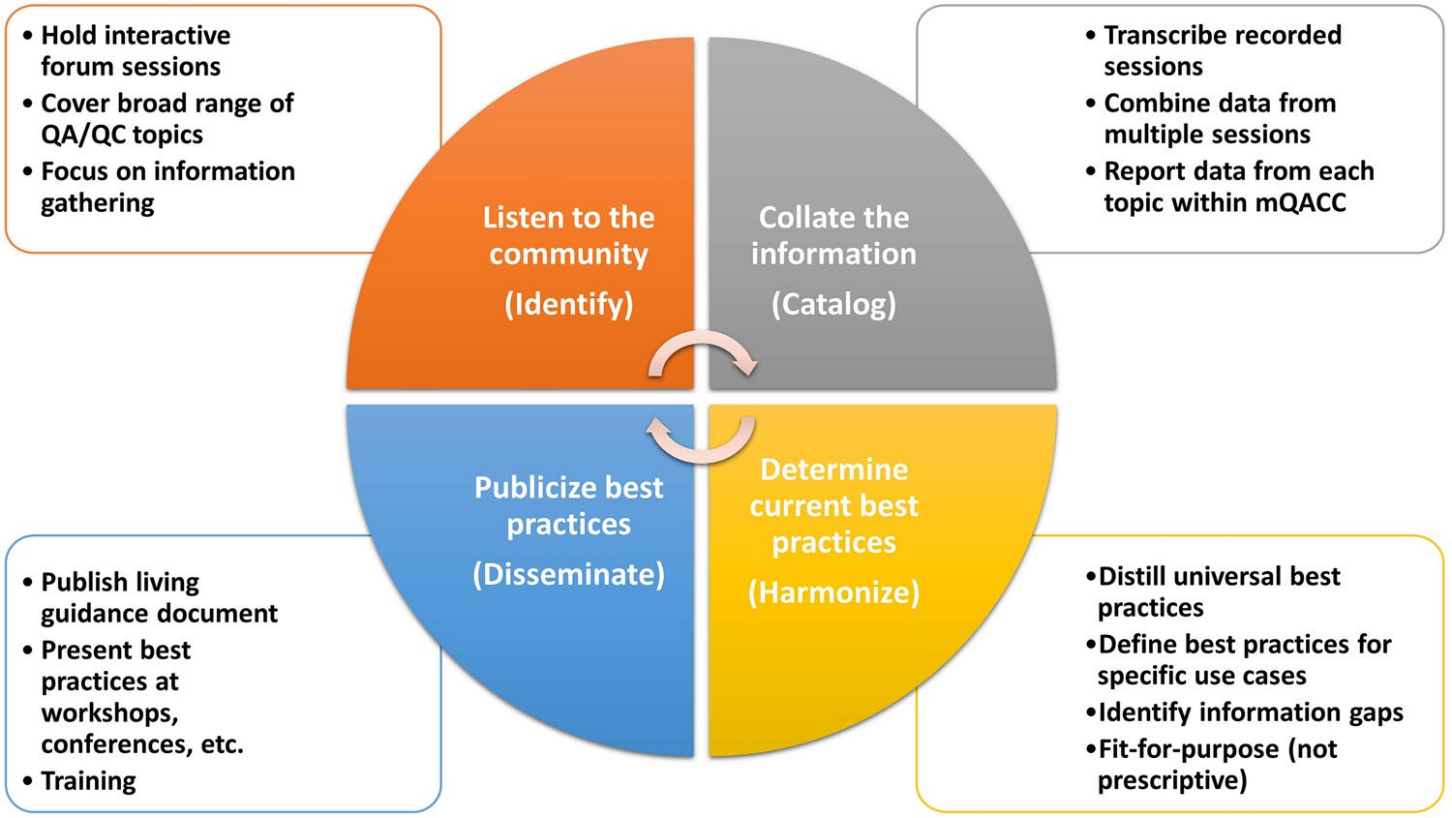
Roadmap for how the Best Practices WG within mQACC is meeting the defined mission statement

**Fig. 2:**
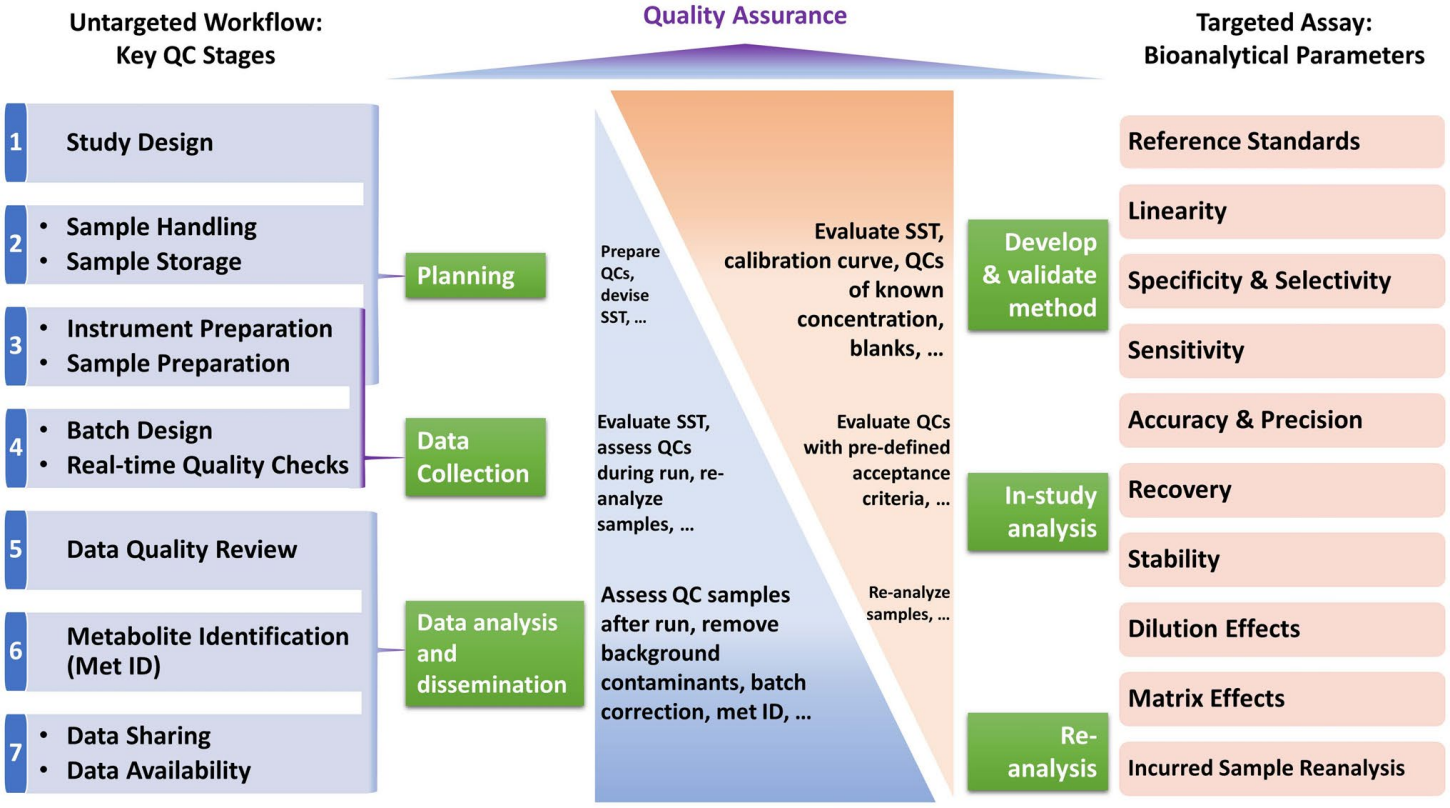
QA and QC practices for an untargeted metabolomics workflow, applied across seven key stages in a retrospective manner, contrasted with the prospective method validation necessary to establish bioanalytical parameters for targeted chromatographic assays (FDA, 2001; FDA, 2018)

It is the mission of mQACC to engage with the metabolomics community and to communicate and promote the development, dissemination, and harmonization of best QA/QC practices in untargeted metabolomics. Two specific consortium objectives that directly pertain to the Best Practices WG are to identify, catalog, harmonize and disseminate QA/QC best practices for untargeted metabolomics (Fig. 1 and 2) and to establish mechanisms to enable the metabolomics community to adopt QA/QC best practices. In order to meet these objectives, the Best Practices WG has developed a working strategy, or roadmap (Fig. 1), that will guide actions and progress, and forge a direction toward use of accepted metabolomics QA/QC practices.

### Roadmap

*Listen to the community*. Best practices are built on tried-and-true practical experiences that demonstrate reproducibility in an untargeted assay. Protocol consensus is a grassroots, community-driven, endeavor with justifications stemming from the impact of methodological successes and challenges. Thus, our process is focused on information gathering from the metabolomics community at large, covering a broad range of QA/QC topics with the goal of being inclusive of all analytical platforms (e.g., LC-MS, GC-MS, and NMR spectroscopy, among others). Activities include conference workshops, interactive forum discussions held through mQACC and platforms that are open to the broader community, and community surveys (see Table 1 for a list of all community engagement activities held to date by the Best Practices WG). All community engagement activities have consisted of sets of polling questions answered by most, if not all, participants. Workshops and forums also included a guided discussion period, where all audience members were encouraged to participate in a deeper discussion about the polling results and other pressing issues.

**Table 1:** Information-gathering activities conducted by the mQACC Best Practices WG from 2019 to 2023

Event	Date	QA/QC Topic	Approximate No. of Participants
1st annual MANA conference workshop	November 16th, 2019	Use of Pooled QCs in LC-MS-based Untargeted Metabolomics	30
European RFMF Metabomeeting 2020 community survey	January 22nd – 24th, 2020	Use of Pooled QCs in LC-MS-based Untargeted Metabolomics	30
mQACC-HHEAR virtual meeting interactive forum (part 1)	June 19th, 2020	Use of Pooled QCs in LC-MS-based Untargeted Metabolomics	15
mQACC-HHEAR virtual meeting interactive forum (part 2)	July 14th, 2020	Use of Pooled QCs in LC-MS-based Untargeted Metabolomics	15
2nd annual MANA conference virtual workshop	September 14th, 2020	System Suitability Evaluation prior to LC-MS-based Untargeted Metabolomics	25
mQACC virtual interactive forum	February 23rd, 2021	System Suitability Evaluation prior to LC-MS-based Untargeted Metabolomics	45
mQACC virtual interactive forum	April 29th, 2021	Use of Internal Standards in LC-MS-based Untargeted Metabolomics	45
mQACC virtual interactive forum	June 14th, 2021	Design of the Analytical Batch in LC-MS-based Untargeted Metabolomics	30
mQACC virtual interactive forum	November 30th, 2021	Quality of Metabolite Annotation & Identification in LC-MS-based Untargeted Metabolomics	35
mQACC virtual interactive forum	March 10th, 2022	Use of Reference Materials in LC-MS-based Untargeted Metabolomics	25
mQACC virtual interactive forum	May 26th, 2022	Data Quality Review in LC-MS-based Untargeted Metabolomics	20
18th annual conference of the Metabolomics Society workshop	June 19th, 2022	State of QA/QC Best Practices in LC-MS-based Untargeted Metabolomics	190
19th annual conference of the Metabolomics Society workshop	June 19th, 2023	Moving Toward Consensus: mQACC Community Engagement on Best QA/QC Practices in LC-MS-Based Untargeted Metabolomics	100

*MANA *Metabolomics Association of North America, *RFMF* French-speaking Metabolomics and Fluxomics Network, *HHEAR* Human Health Exposure Analysis Resource

*Collate the information*. All forums and discussions are transcribed, live polling and survey questions are tallied, and the Best Practices WG synthesizes all the collected information for each specific QA/QC topic. One result of this exploration is the emergence of key QC stages that follow a logical workflow suffused with a wide range of activities including study design, sample handling and storage, instrument preparation, sample preparation (both study and QC), design of the analytical batch, real-time quality checks, data quality review, and quality of metabolite identification. Here, we can investigate how the community is employing QA and QC in their laboratory practices, what practices lead to confidence in analyte measurement or data analysis/treatment, and what practical challenges are encountered. The accumulated data are then presented back to the mQACC consortium and made available to the public via workshops and conferences, e.g., the Metabolomics Society 2022 and 2023 workshops (Dunn et al., 2023).

*Determine best practices*. These data are being interrogated to direct how the community feedback informs QA/QC best practices. At times, consensus methods (i.e., a general agreement among respondents) are the best practice; however, sometimes the non-consensus is the sounder scientific procedure. We propose to distill universal best practices (e.g., use of pooled QCs), isolate best practices for special use cases (e.g., analytical batch design for large cohort studies), identify information gaps (e.g., the lack of an accepted approach for the use of internal standards in quality control procedures) and describe controversial/disputed procedures (e.g., objective parameters to evaluate chromatographic peak shape or evaluate pooled QC repeatability). Most importantly, current best practices should be bound by the “fit-for-purpose” principle with its inclusive, flexible, and non-prescriptive nature.

*Publicize best practices*. To disseminate the best practice guidance for quality control of untargeted metabolomics, we intend to publish a living guidance document, inspired by the FDA’s Bioanalytical Method Validation report (FDA, 2001; FDA, 2018), which can be periodically updated with continued community feedback. In addition, the results will be presented at community-focused convocations such as workshops and conferences to encourage widespread adoption of current best practices and extend the discussions, encouraging the evolution of metabolomics QA/QC best practices. This will include both in-person and virtual events, social media outreach as well as publications to ensure wide dissemination to all practitioners and users of metabolomics. Furthermore, mQACC is actively engaging with journals and data repositories and is providing community resources to promote the accurate reporting of QA and QC practices applied in untargeted metabolomics research (Kirwan et al., 2022). In addition, mQACC will continue to engage instrument vendors, both through membership in mQACC and direct engagement, to relay the importance of incorporating various QC best practices into their software including real-time monitoring and long-term historical QC monitoring. By engaging with the community at all levels, mQACC is ensuring that best practice guidance is as widely adopted as possible.

Over the past few years, the mQACC Best Practices WG has had tremendous momentum and has successfully collected and collated information from 13 sessions involving more than 600 participants in total (see Table 1). At present, the focus of the WG is on advocating QA/QC practices, gathering information and feedback from the community and introducing a living guidance document to help cultivate the undeniable potential of metabolomics.

### Guiding principles

Creation of a focused resource with comprehensive guidance for QA/QC in untargeted metabolomics experiments is the overarching purpose of the mQACC Best Practices WG. Specifically, the aim is to ensure the production of high-quality, reliable data that enable confidence in study results and promotes intra- and inter-study reproducibility. Given that QC practices for untargeted profiling are retrospective, in contrast to a validation process for targeted analysis, untargeted QC necessitates the incorporation of additional design and best practices to achieve the stated goal (Fig. 2). Here, we establish the framework for a QC living guidance document which will follow the typical phases of a metabolomics experimental workflow (planning, data collection, data analysis and dissemination) with guidance of QC activities at key QC stages throughout the workflow (see Table 2). Importantly, this guidance will also incorporate and expand upon the terminologies and examples recently published by mQACC (Kirwan et al., 2022). For ease of reference, this glossary is reproduced in the Supplemental File.

### Metabolomics research framework

*Planning phase.* Activities occurring prior to measurement of the metabolome set the stage for a successful research study by assuring quality procedures are determined and implemented (Broadhurst et al., 2018). For instance, defining the scope of the research study can include (but is not limited to) formulating the research problem, determining the purpose of the experiment, and reviewing relevant literature. Untargeted metabolomics is often a hypothesis-generating (inductive) venture, while deductive experiments could be considered in this phase as well (e.g., whether to incorporate targeted analyses to test relevant hypotheses or use the emerging integrated targeted-untargeted approaches). Ideally, all parties involved will have a seat at the table when planning the endeavour. Metabolomics is, in most cases, a team effort; expertise and agreement among the biology/clinical/plant/microbial/environmental scientists, analytical chemists and statistician/bioinformatic/epidemiology scientists is paramount. **Study design** is of key importance to a metabolomics project (Fig. 2; Table 2). At this stage, protocols will be developed for the collection, preparation, and analytical testing of QC materials, such as pooled QCs and process blanks. Additional QC materials are often necessary, for example internal standards or reference materials, and discussion regarding acceptance criteria and data analysis practices will define the requirements for confident analysis of high-quality data pertinent to the specified research question. **Sample handling and storage conditions**—including shipping—can affect the integrity of the sample, contributing unwanted variation in the intended metabolome and influencing the proposed conclusions away from the study objective (Dudzik et al., 2018). Important aspects to consider include sampling technique, time of collection, timeframe from sampling to quenching of metabolism, metabolism quenching method, and optimal transport/shipping and storage temperatures for assuring sample stability. In the context of preparation of materials, considerations must be made for the method of extraction of metabolites, selection of internal standards, the type and composition of system suitability test samples, blank samples, and reference materials. **Instrument preparation** is another key aspect of the planning phase that encompasses steps for instrument preparedness, optimization of methodologies in the context of the experimental sample composition, and SST, which covers all activities performed prior to analysing any study sample to demonstrate the analytical system is fit-for-purpose and working within specifications. The main goal is to gauge the performance of the instrument by evaluating system accuracy and precision and to identify extraneous contaminants, taking remediation actions if warranted (Broadhurst et al., 2018). LC-MS relevant activities include both general and assay-specific SST activities such as chromatographic resolution, mass calibration and/or mass accuracy check, analysing blank samples and SST samples; however, guidance will eventually be expanded to include activities for all major instrument platforms. Likewise, the **sample preparation** process should be considered during the planning phase, driven by the fit-for-purpose principle. Sample preparation can be problematic due to inserting unwanted variance with manual missteps (Vuckovic, 2012), necessitating its inclusion as a key QC stage. Overall, many facets must be defined and attended to during the planning phase including, but not limited to, format (vials or plates), structure (batching or block), extraction order, replicates, study-specific aspects, blanks, intra-study QC samples, internal standards, and reference materials. As necessary, the guidance will define terms such as nomenclatures contrasting different types of blank samples (process blank, solvent (true) blank, etc.), and will describe use strategies for a variety of instrument and sample preparation scenarios (see Supplemental File).

**Fig. 3:**
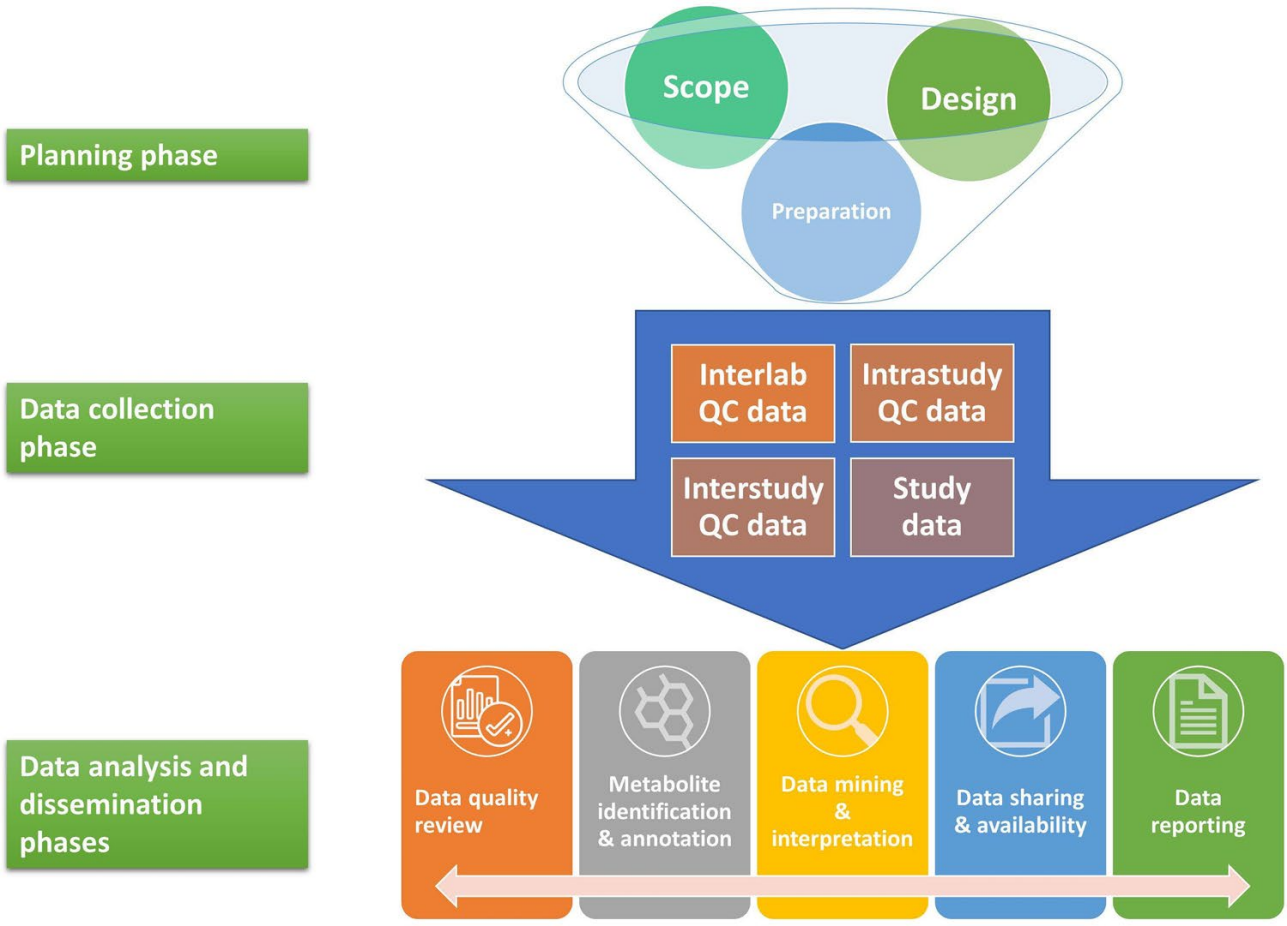
A proposed framework for the living guidance document that mQACC plans to publish. Initial guidance will be grouped into three main phases dictated by the basic tenets of a metabolomics research study. As additional considerations develop, they can be incorporated into any of these phases where appropriate. Thus, the guidance can grow along with the field

*Data collection phase*. The data collection phase contains a plethora of introduced experimental variation from multiple sources, thus demanding consideration of QA/QC practices in safeguarding the resultant metabolome measurements and minimizing loss of precious samples. Hence, the data collection phase encompasses the gathering of all metabolomic data required to address the research problem in a reliable manner, which should include QC data in addition to study data (Fig. 3; Table 2). In line with the preparatory aspects discussed above, a stringent **batch design** is necessary to best avoid instrument/system fluctuation and minimize the need for data treatment post-collection (see Supplemental File for the definition of a batch). This is especially a concern for large-scale studies that necessitate consistency in data generation over days, weeks, months, and even years. QA/QC topics for contemplation should comprise details of the blocking and order of samples within a batch (randomization, study factor orthogonalization, interleaving, etc.), SST samples, system conditioning samples, MS/MS parameters, dilution series for linearity assessment, and placement and frequency of QC samples, blanks, and reference materials. Additionally, the use of technical replicates (preparation and/or injection) should be considered where they suit the selected study design. The use of **real-time quality checks** (i.e., before a batch is completed) will determine the types of interventions within a batch (e.g., decide whether to stop or intervene with the batch), including the relevant metrics to use and criteria for reinjecting a failed sample. This stage is possible with the inclusion of internal standards in the metabolomics workflow. Importantly, the collection phase should be an iterative process which allows for the re-generation of study and QC data, when necessary. Therefore, guidance will also include use strategies for a variety of batch designs and data (re)generation scenarios.

*Data analysis and dissemination phases*. Unlike with the planning and data collection phases, where one funnels information into the next in a unidirectional manner (Fig. 3), the data analysis and dissemination phases inform each other via a bilateral flow of information. With a substantial amount of complex metabolomic data in-hand, one must evaluate the quality of the data and determine processing practices that yield only well-measured features (i.e., signals that comply with previously established criteria) that are to be analysed in the context of the original study objective. Quality checks can begin immediately as the data is generated in a real-time appraisal of the system. However, many critical aspects of **data quality review** can only be accomplished once data collection is complete. For instance, pooled QCs require a post-hoc assessment when the full data set can be processed as one in a like fashion to enable a formal analysis of the created data matrix (Broadhurst et al., 2018). Other QC materials valuable to data quality review include process blanks, reference materials (e.g., long term QC), and phenotypic pooled QC samples (see Supplemental File). Data quality metrics for QC samples can vary based on the analytical platform used, and so platform-specific metrics will be included in the guidance document. With LC (or GC)-MS-based metabolomic workflows, such metrics can assess chromatographic (retention time (t_R_) drift, peak shape, and chromatographic resolution) and mass spectral (*m/z* accuracy and signal response) fluctuations, as well as batch and run-order effects. Of course, some aspects of data quality review are also independent of the analytical platform. For the analysis and dissemination phases, the guidance document will inform on both universal and specific-use case best practices for data analysis methodologies that explain what to do when QC samples fail acceptance criteria, how to detect sample outliers, how to manually check data, how to use data obtained from blanks and steps to take to improve data quality. Another important concept to note here is redundancy in QC strategies. The extra investment to include multiple QC sample types is worthy of consideration and may save an expensive and unrepeatable research study.

Quality of **metabolite identification/annotation** is fundamental to the particular purpose of understanding the mechanistic role of biochemical constituents contributing to a specific phenotype and fostering metabolic biomarker discovery. Metabolite identification has been a persistent bottleneck in MS-based metabolomics research predominantly due to the type and volume of data used and the availability and quality of the retention time and mass spectral libraries where the number of chemical standards used to construct these libraries is small compared to the size of sample-specific metabolomes. NMR spectroscopy-based metabolite annotation has its own challenges with respect to metabolite identification and will be addressed in later versions of the guidance (Wishart et al., 2022). Regarding the former, commonly used data types include MS^1^ and MS^2^ (MS/MS) spectra and t_R_ data, currently with less frequent use of MS^n^ spectra and ion-mobility collisional cross-section (CCS) data. Computationally, annotation/identification quality depends upon use of MS/MS and t_R_which relies on the use of libraries based on authentic standards, either publicly accessible or created in-house, or with use of publicly available MS/MS in-silico libraries. Library curation, longevity (of both software and library), compound enumeration and coverage, and search algorithm are important factors when considering an identification tool. In conjunction with reporting the Metabolomics Standards Initiative (MSI) confidence level (Sumner et al., 2007), providing evidence of confidence is important to mitigate high false discovery rates for metabolite identification. Such evidence can be determined by manual evaluation of quality indicators like *m/z* and t_R_ error, MS/MS or MS^n^ match score and isotopic distribution match score. For true confidence in a metabolite identification (Level 1), the MSI suggests identity by two orthogonal properties, in conjunction with a match to an authentic standard run under the same analytical conditions (Sumner et al., 2007), a practice which is applied only to approximately 20% of the published works (Kodra et al., 2022). Importantly, a lack of reporting the evidence of confidence in metabolite annotation can severely limit the ability to assess the veracity of data mining and interpretation results for a given study. While no information gathering events specifically regarding these aspects of data mining and interpretation have been held to date (see Table 1), we recognize this is an important area regarding QC practice and plan to address it in the next iteration of the roadmap (Fig. 1).

Another QC stage for which we have not yet gathered information but nevertheless have considered important to include in future versions of guidance, encompasses the sharing of scientific data. This key stage allows use and re-use of generated data contributing to an enhancement in the scientific impact of metabolomics. Indeed, existing policy from the National Institute of Health dictates the inclusion of **data sharing and availability** in the metabolomics workflow (National Institutes of Health (NIH), 2023). Publicly available data, such as those found in the Metabolomics Workbench and/or MetaboLights (2023b); (Haug et al., 2020), can potentially aid in more efficient, well-founded discoveries of molecular markers of disease/exposure or environmental status, leading to subsequent interventions, regulations and mitigation strategies. Additionally, access to high-quality data that is properly reported with all necessary information provides the foundation for advancing software and informatic tool development, highlighting the need for data reporting standards both for data deposition and publication (Kirwan et al., 2022). For instance, accessible data for pooled QCs, blanks and reference materials will promote (1) confidence in the research study, (2) interlaboratory reproducibility, and (3) harmonization and community consensus for protocols, methodologies, measurement robustness of specific analyte presence and quantities, and integration of multi-omics data sets, to help address the current challenges facing the field. This important aspect of the data analysis and dissemination phases will be strongly encouraged as an influential gearwheel that drives metabolomics progress. However, the sharing of data for QC samples in metabolomics data repositories is currently very limited.

### Avenues to contribute

Information gathered from experienced and knowledgeable metabolomics practitioners will form the basis of the QA/QC living guidance document. While mQACC is actively pursuing avenues for community engagement, practical expertise among metabolomics practitioners is critical to encourage community adoption of current QA/QC best practices. We therefore call on wider metabolomics, lipidomics, and exposomics communities to contribute to these efforts on an on-going basis. **Possible avenues to contribute**: **1**) **Lend your voice** and guide identification and cataloguing of practices via active participation in surveys, workshops, forums, and discussions. The roadmap (Fig. 1) is in its first cycle and the development of successful universal guidance requires on-going participation from practicing scientists across diverse application areas in successive iterations. **2**) **Report QC practices in manuscripts** in a detailed and standardized manner (Kirwan et al., 2022; Wilson et al., 2021). Provide the detailed descriptions that are essential to produce repeatable results, preferably using checklists developed by mQACC for this purpose (Kirwan et al., 2022). Show data before and after filtration and processing for transparency and understanding. Report acceptance criteria for QCs. **3**) **Require fit-for-purpose quality measures** when acting as a reviewer or journal editor. If QC practices are not implemented or adequately described, one should request their inclusion and re-review in a resubmitted work, otherwise it is impossible to attest to the validity of the generated data. Considering the value of publications for scientific funding and professional success, this request from reviewers and journal editors is likely to be one of the driving forces that encourages the community to adopt QA/QC best practices. **4) Become a member of mQACC** and actively engage in its mission to communicate and promote the development, dissemination, and harmonization of best QA/QC practices in untargeted metabolomics. To see how you can get involved, seek out a current mQACC member and consider submitting an application (Metabolomics 2023a).

## Final thoughts

Metabolomics is an increasingly integral component of multidisciplinary science. As stated above, the community is in dire need of accepted QA/QC practices to implement the newly developing (and existing) technologies and methodologies that comprise the field as a whole. Employing best QA/QC practices will enable, and perhaps accelerate, the evolution of the field. To this end, it is critical that the community not only develops the proposed guidance, but also does so in such a way that the information grows and adapts as the QA/QC practices themselves change with the field. This is the reason that we propose a framework to establish a living guidance document (Fig. 3). Indeed, this framework allows for inclusion of new topics, like how the Best Practices WG is currently planning the addition of data mining and interpretation guidance after completing the first iteration of the roadmap (Fig. 1; Table 2). Consistent with ongoing mQACC planning efforts, the final guidance will comprise a version-controlled, open-access website—accompanied by a peer-reviewed journal publication—that allows for well-defined, periodic editing of its content and structure. Guidance will include access to full and summary datasets of mQACC information-gathering efforts, example case studies and links to relevant literature, didactic documents explaining various QC approaches and which aspects they address, and checklists for authors, reviewers and journal editors to consult for publications. The initial version will be agreed and reviewed by mQACC and incorporate the results of extensive 5-year community engagement. Once publicly available, the website will have a dedicated section to submit comments, suggestions and requests for revision/addition. Importantly, this will remain a community-based effort where input will be continually sought. This feedback will be reviewed by a dedicated committee of experts set up by mQACC and incorporated periodically into new version(s) of guidance. Thus, the website allows for collection of feedback, in addition to periodic editing, as QA/QC practices are further refined. It also allows for inclusion of training materials and linking to other QA/QC resources beyond mQACC to help the wider community of users and practitioners during selection and implementation of fit-for-purpose QA/QC strategies for their study. By establishing this framework for best QA/QC practices for untargeted metabolomics, we endeavor to forge a path towards its widespread acceptance. For this to be successful, however, members of the community must continue to participate in its inception, dissemination, adoption, and evolution.

**Table 2:** Hierarchical metabolomics research framework underpinning the proposed living guidance document

Phase	Subphase	Examples from metabolomics workflow
*Planning* Encompasses scope, design and preparation of materials, instrument, study samples and quality control (QC) samples	Scope	• Formulating the problem • Determining the research purpose • Literature review • Determining the hypothesis (if applicable)
	Design	• Selecting a study design • Developing the instrumental method fit for purpose to the chosen research problem o Analytical platform o Platform-specific details ■ LC-MS (columns, gradients, additives, etc.) ■ GC-MS (columns, gradients, derivatization, etc.) ■ NMR (field strength, internal chemical shift standard, solvents, etc.) ■ Other • Determining sampling, handling, shipping and storage • Determining sample processing and extraction • Planning for data collection (blocking, order, technical replicates, etc.)
	Preparation	• Of materials: o Internal standards o System suitability test (SST) samples o Blank samples (true, process) o Reference materials ■ Long term QC samples ■ Certified reference material samples ■ Standard reference material samples • Of instrument: o Calibration o SST o Running blanks o Updating logbooks, etc. • Of study and QC samples: o Sample processing and extraction (can include spiking of IS) o Process blanks o Pooled QC samples ■ Intrastudy ■ Phenotypic o Sample reconstitution (can include spiking of IS)
*Data collection* Encompasses the gathering of all data necessary to address the research problem in a reliable manner	Batching & Blocking	• Header & footer o SST o Blanks o Conditioning QCs o MS/MS o Periodic injection of QCs o Reference materials o Dilution series, etc. • Sample blocks
	Order	• Randomization • Study orthogonalization • QC sample intervals
	Technical replicates	• Preparation replicates • Injection replicates
	Real-time quality checks	• Within batches o Metrics to use o Acceptance criteria • Between batches o Metrics to use o Acceptance criteria
*Data analysis and dissemination* Encompasses the evaluation of the quality of the metabolomics data generated in terms of the reliability of the qualitative and relative quantitative measurement of the metabolomic profile.	Data quality review	• Accuracy and/or precision of overall method • Materials & order of analysis o Blank samples o Long- and short-term reference samples o Pooled QCs o Internal Standards o Test mixture solutions, etc. • Metrics o *m/z* accuracy o intensity/signal response o retention time drift, etc.
	Metabolite annotation & identification	• Type and quality of data used for ID • Evidence of confidence • Quality of computational resources used
	Data mining & interpretation	• Type of statistical analysis • Pathway analysis, if applicable
	Data sharing & availability	• Deposit QC and sample data in repositories • Metadata with clear description of QC sample types
	Data reporting	• See mQACC recommended reporting guidelines (Kirwan et al. 2022)

See Glossary in Supplemental File for definitions with specific examples of terms used to describe various aspects of a metabolomics workflow

### Supplementary Information

Below is the link to the electronic supplementary material.

Supplementary material 1 (PDF 202.2 kb)

## Data Availability

All data are included in the manuscript.
