# Supplementary material for: Establishing a framework for best practices for quality assurance and quality control in untargeted metabolomics
Source: Metabolomics. 2024 Feb 12;20(2):20. doi: 10.1007/s11306-023-02080-0 (PMC10861687; doi:10.1007/s11306-023-02080-0)
Supplement: Supplementary file 1 — Supplementary material 1 (PDF 202.2 kb) [file 11306_2023_2080_MOESM1_ESM.pdf]

On acceptable practices for quality assurance and quality control in untargeted metabolomics

Journal Name: *Metabolomics*

Jonathan D. Mosley*, Tracey B. Schock, Chris W. Beecher, Warwick B. Dunn, Julia Kuligowski, Matthew R. Lewis, Georgios Theodoridis, Candice Z. Ulmer Holland, Dajana Vuckovic, Ian D. Wilson, Krista A. Zanetti, on behalf of the Metabolomics Quality Assurance and Quality Control Consortium (mQACC)

* Corresponding author

Email: mosley.jonathan@epa.gov

Affiliation: Center for Environmental Measurement and Modeling, Environmental Protection Agency, Athens, GA 30605, USA

Glossary[a]

Term	Definition
Precision	Performance characteristic of the analytical procedure that is applied to produce the untargeted spectral profiling data.<br><br>It indicates the typical measurement variability of the repeated measurements of the same sample within a time period of a batch (repeatability) or over longer periods e.g., between batches (intermediate precision) and over a long period of time (intra-laboratory reproducibility).<br><br>It can be assessed by designed replicate analysis of QC samples under the operational conditions and it is usually expressed by statistical parameters which describe the variability of the data e.g., the standard deviation or relative standard deviation of signals.<br><br>Accuracy is not the same as precision as, unlike the latter, it cannot easily be determined in untargeted MS-based profiling as the identities and quantities of the solutes in the sample are not known prior to analysis.
Mass accuracy	Mass Accuracy is a measure of how close the measured mass of a standard calibrant is to that obtained when measured

	experimentally. It is an important measure of mass-spectrometric instrument performance.
Intra-laboratory reproducibility or Within-laboratory reproducibility	Indicates the variation in the analytical data if the same sample is analyzed in the same laboratory at different times. It encompasses the whole analytical process from the sample entering the laboratory to the report.
Inter-laboratory reproducibility or Between-laboratory reproducibility	Indicates the variation in the analytical data if the same sample(s) are analyzed in different laboratories.
Batch	The collection of experimental samples, and QC samples (including, but not limited to blanks, experimental sample replicates and other pertinent quality-related samples) from a study that are processed at one time and analyzed in a single, non-stop instrumental run.
QC sample(s)	Various types of mixtures prepared to assess the quality of untargeted data.
Pooled QC sample	A matrix-matched QC sample prepared by mixing aliquots from all the samples of a study. In the case of large studies, it can be prepared from a subset of the samples. Various types of pooled QC can be prepared depending upon their intended use (see below).
Phenotypic pooled QC sample	When a matrix-matched phenotypic QC for each class of samples under study are prepared separately. It can be used to highlight differences in precision in test vs control samples for signals differentiating between the classes.
Intra-study QC sample (Including Intra-batch/Within-batch QC and Inter-batch/Between -batch QC)	A QC sample that is used to assess the precision of untargeted data along a single batch (intra-batch QC sample), or a single study (inter-batch QC sample). It can be a pooled QC prepared from aliquots of the samples analyzed in the batch itself (see above) or, where this is impractical, other approaches (e.g., a "bulk" QC sample of the same matrix) can be used.
Long term QC (can be subdivided into intra-lab /within-lab and inter-lab/between-lab)	A reference material or a bulk QC sample prepared from a set of samples, or a bulk sample/reference material purchased from a public source. It can be used to assess analyses undertaken over a relatively long period for intra- and inter laboratory reproducibility. It can be used to assess (and potentially correct for) any differences between separate studies on the same type of sample. Intra-laboratory (within-lab) QC samples are used only within a single laboratory. Inter-laboratory (between-lab) QC samples are used to compare data between two or more labs.
Blank(s)	Blank QC samples are important in demonstrating process purity, where extraneous signals are identified and accounted for. A true blank is a neat solvent/buffer etc., with minimum pre-processing, that is directly analyzed by the instrument. If the sample is a buffer aliquot, for example, it could be either a

	process blank or a true blank, depending on how it is handled. A process blank consists of a neat solvent, water, buffer etc., sample processed in exactly the same way as the samples. It can be used to examine the interferences or contamination introduced by the analytical system, columns, vials or the sample preparation step.
Test mix(ture)	Often a synthetic mixture of metabolites, including representatives of the metabolites expected to be in the study samples. It can also be a mixture of exogenous compounds or xenobiotics that are easily or often detected in the analytical system used.
System suitability test (SST)	Analysis(es) performed before the analytical batch of the study samples to check that the analytical system is working appropriately and fit-for-purpose. It can be performed using QC samples or with a specific standard mixture (see above).
Reference materials	A reference material is a material which is sufficiently well characterized, homogenous and stable to be fit for its intended use in a measurement process. Reference material is a generic term. A certified reference material (CRM) has been additionally characterized by a metrologically validated procedure for specific characteristics which will be documented in an accompanying certificate including the allowable measurement uncertainty. A standard reference material (SRM) is a reference material certified to particular requirements laid down by the US National Institute of Science and Technology (NIST).

[a]Reproduced without changes (Kirwan *et al.*, 2022). To view the license of the original work, visit http://creativecommons.org/licenses/by/4.0/

Reference

Kirwan, J.A., Gika, H., Beger, R.D., Bearden, D., Dunn, W.B., Goodacre, R., Theodoridis, G., Witting, M., Yu, L.R., Wilson, I.D., metabolomics Quality, A. and Quality Control, C. (2022) Quality assurance and quality control reporting in untargeted metabolic phenotyping: mQACC recommendations for analytical quality management. *Metabolomics* **18,** 70.
